# Structure and Performance Attributes Optimization and Ranking of Gamma Irradiated Polymer Hybrids for Industrial Application

**DOI:** 10.3390/polym14010047

**Published:** 2021-12-23

**Authors:** Suhail H. Serbaya, Emad H. Abualsauod, Mohammed Salem Basingab, Hatim Bukhari, Ali Rizwan, Malik Sajjad Mehmood

**Affiliations:** 1 Department of Industrial Engineering, Faculty of Engineering, King Abdulaziz University, Jeddah 21589, Saudi Arabia; sserbaya@kau.edu.sa (S.H.S.); mbasengab@kau.edu.sa (M.S.B.); 2 Industrial Engineering Department, College of Engineering, Taibah University, Al MadinaAlmonawara 41411, Saudi Arabia; eabualsauod@taibahu.edu.sa; 3 Department of Industrial and Systems Engineering, College of Engineering, University of Jeddah, Jeddah 21959, Saudi Arabia; habukhari@uj.edu.sa; 4 Department of Basic Sciences, University of Engineering and Technology, Taxila 47050, Pakistan; msajjad.82@gmail.com

**Keywords:** polymer modifications, polymer ranking, properties optimization, UHMWPE, graph theory, UHMWPE/silane hybrids, radiation modifications

## Abstract

The selection of suitable composite material for high-strength industrial applications, from the list of available alternatives, is a tedious task as it requires an optimized structural performance-based solution. This study aimed to optimize the concentration of fillers, i.e., vinyl tri-ethoxy silane and absorbed gamma-dose, to enhance the properties of an industrial scale polymer, i.e., ultra-high molecular weight polyethylene (UHMWPE). The UHMWPE hybrids, in addition to silane, were treated with (30, 65, and 100 kGy) gamma dose and then tested for ten application-specific structural and performance attributes. The relative importance of attributes based on an 11-point fuzzy conversation was used for establishing the material assessment graph and corresponding adjacency matrix. Afterwards, the normalized values of attributes were used to establish the decision matrix for each alternative. The normalization was performed after the identification of high obligatory valued (HOV) and low obligatory valued (LOV) attributes. After this, suitability index values (SIVs) were calculated for ranking the hybrids that revealed hybrids 65 kGy irradiated the hybrid as the best choice and ranked as first among the existing alternatives. The major responsible factors were higher oxidation strength, a dense cross-linking network, and elongation at break. The values of the aforementioned factors for 65 kGy irradiated hybrids were 0.24, 91, and 360 MPa, respectively, as opposed to 0.54, 75, and 324 MPa for 100 kGy irradiated hybrids, thus placing the latter in second place regarding higher values of Yield Strength and Young Modulus. Finally, it is believed that the reported results and proposed model in this study will improve preoperative planning as far as considering these hybrids for high-strength industrial applications including total joint arthroplasty, textile-machinery pickers, dump trucks lining ships, and harbors bumpers and sliding, etc.

## 1. Introduction

Ultra-high molecular weight polyethylene (UHMWPE) is a versatile engineering material for several high-strength industrial applications [1,2]. To enhance its strength and improve oxidation stability, the cross-linking of UHMWPE is conducted either chemically and/or with gamma rays [3]. Although, the strength of highly cross-linked UHMWPE is much higher as compared to pristine ones, the issue of the long-term oxidation stability of highly cross-linked UHMWPE persists due to the formation of free radicals during the process of irradiation. To cope with the aforementioned issue, the inclusion of α-tocopherol (vitamin E) is proposed, but the competitive role of α-tocopherol during the cross-linking reaction is responsible for reducing the cross-linking yield of radiation treatment. This reduction in cross-linking yield led the way for UHMWPE/silane hybrids, where double cross-linking yield for the gamma-irradiated hybrid can be obtained as compared to pristine UHMWPE. Furthermore, the oxidation and thermal resistance of 65 kGy irradiated hybrids are found superior to water cross-linked hybrids, 30 kGy, and 100 kGy irradiated ones. However, the yield strength and Young’s modulus of the 100 kGy irradiated hybrid are higher as compared to others [2,3,4,5,6]. In short, the aforementioned approach is proved to be a new alternative; however, an optimized solution based on the structural and performance attributes is required for choosing the best alternative from the group of water treated and gamma-irradiated hybrids. This is because some structural properties are better for one alternative, and performance properties are better for other alternatives.

In the past, the optimized solution based on the structural and performance attributes of the polymer composites were usually simulated experimentally while testing them on wear–tear testing apparatus [7]. The experiments which were used for simulating the performance include accelerating aging, testing the samples on application-specific simulators, etc. [1,8]. However, such multi-attribute optimization problems can also be dealt with by introducing the suitable material selection model. This approach is equally useful for avoiding the misuse of resources and saving time and cost [9,10,11]. Furthermore, using material selection models to select, sort, and prioritize the alternatives on the optimized structure–properties relationship (while keeping in view the requirements of the end-user) provide a flexible approach for a wide range of applications. The material selection models are usually based on more than one attribute for the final decision concerning optimum choice. The information regarding the structural, physical, chemical, mechanical, thermal, and morphological properties of the material used is therefore required for the final product design [12,13,14,15]. There are various material selection models proposed by many researchers but few with satisfactory results as far as their utility to the subject matter of interest:

- The material selection model based on fuzzy multi-attribute decision-making, proposed originally by Liao [16], is rather complex in nature and requires significantly large computational time.
- The model based on the ‘utility’ functions for multi-objective material optimization proposed by Ashby [17] is simple, but its effectiveness is questionable.
- A material selection model known as ELECTRE, based on multi-attribute decision-making, uses the theme of outranking-relationship among the attributes [18] but can only compute the partial prioritization among small numbers of alternatives.
- Bahraminasab and Jahan [19] recently used the comprehensive VIKOR model for the selection of a femoral-component for TKR. The results are satisfactory to some extent; however, this study is site-specific and needs to be amended for consideration at other replacement sites.

For industrial applications of high-strength polymers, the MCDM approach has already been adopted by the scientific and industrial community for obtaining the optimal formulation. For example, Mahesh et al. [20] recently used this method to select the optimum polymer fibrous structure for impact load applications. In another study, the formulation of making polyester/chopped glass fiber composite was optimized using the multi-attribute decision-making approach [21]. In another study, Sharba and Al-Mostaaf [22] used TOPSIS, VIKOR, and AHP to prove that LLDPE and Nylon are the best material for syrup-vessels based on optimum thermal efficacy. However, for UHMWPE and its composites/hybrids, there are few studies in the literature that utilize multi-attribute decision-making for the prioritization of materials for total joint replacement [23,24,25,26]. These studies utilized techniques such as TOPSIS and VIKOR, which are rather complex and require special mathematical and computational skills. Although the application of graph theory for the subject matter of interest is simple and straightforward only a few attempts can be found in the literature. For example, Vecchio et al. [27] have explored its potential while quantitatively assessing the structure of aramid nanofibers. They have utilized SEM micrographs and accurately converted the complex structure of aramid nanofibers into graphs, which were then used for structure analysis with 13 graph-theoretical parameters. In another study, Ahmad A Baksh [28] utilized the concept of graph theory in conjugation with multivariate statistics for selecting optimized UHMWPE composites while considering the structural parameters only, i.e., oxidation strength, percent crystalline, and cross-linking yield. Unfortunately, the fundamental mechanical characteristics (elongation at break, Young Modulus, and Yield strength that are the required performance attributes for utilizing UHMWPE and its composites in any industrial application) were not considered. Moreover, thermal characteristics, i.e., thermo-oxidative activation energies and thermal degradation energies (which are also associated with the performance efficiency of UHMWPE-based materials) were also not considered. Another major limitation of the study is that the reported computational results relied solely on one experimental investigation, i.e., FTIR analysis, thus making the authenticity of the results questionable. To the best of our knowledge, there is no such model proposed to date that has focused on structure–property optimization before and after any modification and/or designing (radiation sterilization and modifications) to rank the available alternatives of UHMWPE-based polymer composites.

This paper aims to develop a suitable model for structure–property optimization and ranking the hybrids while using the concepts of graph theory and adjacency matrix. The main motive to develop such a model is to choose the best among the existing alternatives while keeping in view all the important structural and performance attributes (important for industrial applications of UHMWPE and its hybrids), thereby enhancing the service life of hybrids. The technical picture of the manuscript starts while considering theoretical aspects of graph theory and the matrix approach (GTMA) and proposing the methodology for ranking the set of groups, which is subsequently implemented on UHMWPE/silane hybrids. The four hybrids, water cross-linked, 30 kGy, 65 kGy, and 100 kGy, represented by the codes HY_0_, HY_30_, HY_65_, HY_100_, respectively, were considered and tested for different properties for initial treatment. After satisfactory appreciation of the proposed model and a discussion of the best choice, the manuscript ends with conclusive remarks and future recommendations for industrial applications.

## 2. Theoretical Considerations on Optimization Model

The main aim of this study is to introduce the simplest and most robust method for optimizing and ranking the hybrids. The concepts of graph theory are used in this study due to being sophisticated, coherent, simple, and systematical compared to other existing approaches. The progress and implementation of the graph theory concepts for various industrial applications are exceptionally well-defined, where graph/digraph representations have demonstrated to be valuable for modeling and analyzing different systems and issues in various areas of science and technology [29,30]. After the representation of the problem with a problem assessment graph, adjacency matrices are used for the graph/digraph template analysis to quickly deduce the system function and system file to achieve the objectives. Therefore, a graph theory and matrix approach are implemented to establish the structure–property optimization and ranking of hybrids while utilizing the quantitative measure of material attributes. The details of the model are given below:

Step I: Determine the material prioritization factors while considering their importance as far as a subject matter of interest is concerned

F = [F_1_, F_2_, F_3_, F_4_…..F_j_……F_n_] (1)

where F_i_ is the material factor which is either a higher valued obligatory (HOV) or lower-valued obligatory (LOV) factor as far as the nature of the application is concerned.

**Figure 1:**
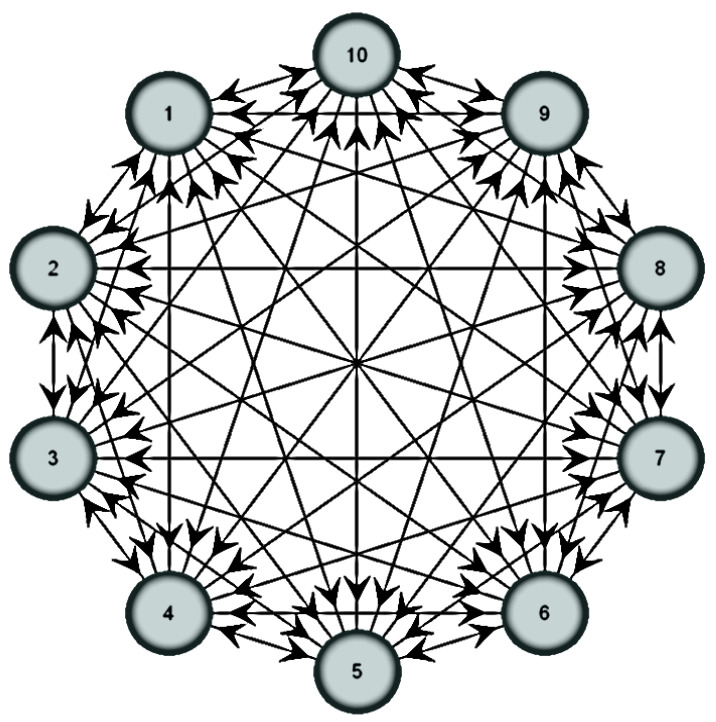
Material optimization attributes graph.

Step-II: Establish the material assessment graph based on the selected material prioritization factor, as represented in Figure 1.

**Figure 2:**
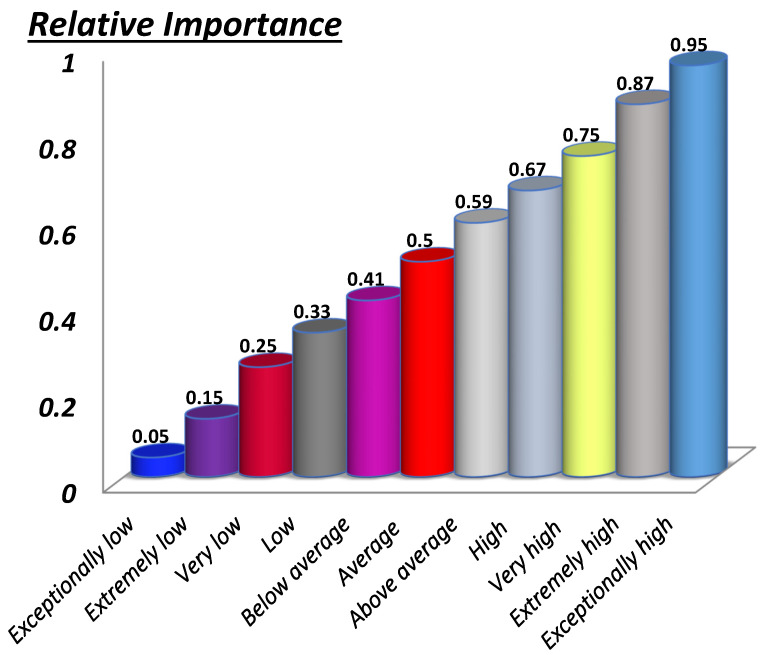
The 11-point fuzzy logic color scale.

Step III: Determine the relative importance of the material prioritization factor (r_ij_) while following the 11-point fuzzy conversion scales [31], which are used to assign verbal relative importance to the corresponding fuzzy numbers. There are other scales available, but in this study, an 11-point scale is used to better represent the relevance of one factor over another as shown in Figure 2.

Step IV: Normalize the quantitative values of each attribute for all four alternatives according to the normalization rules defined for HOV and LOV factors (details are given in the results and discussion section).

Step V: Write the decision matrix for each alternative while writing the adjacency matrix and normalized values of HOV and LOV factors as written below.

(2) $$A=\left[\begin{array}{ccccccccc} Parameter & F1 & F2 & F3 & F4 & F5 & F6 & F7 & F8\\ F1 & R1 & r12 & r13 & r14 & r15 & r16 & r17 & r18\\ F2 & r21 & R2 & r23 & r24 & r25 & r26 & r27 & r28\\ F3 & r31 & r32 & R3 & r34 & r35 & r36 & r37 & r38\\ F4\; & r41 & r42 & r43 & R4 & r45 & r46 & r47 & r48\\ F5 & r51 & r52 & r53 & r54 & R5 & r56 & r57 & r58\\ F6 & r61 & r62 & r63 & r64 & r65 & R6 & r67 & r68\\ F7 & r71 & r72 & r73 & r74 & r75 & r76 & R7 & r78\\ F8 & r81 & r82 & r83 & r84 & r85 & r86 & r87 & R8 \end{array}\right]$$

where Ri is the normalized value of the Fi prioritization factor, and rij is the relative importance of Fi factor from Fj factor while keeping in view the nature of the application.

Step VI: Finally, the suitability index value (SIV) is calculated for each hybrid with the help of the following relation.

SIV = Per (A)(3)

(4) $$\begin{array}{c} \mathrm{Per}\;(A)=\overset{N}{\underset{i=1}{\prod }}R_{i}+\overset{N-1}{\underset{i=1}{\sum }}\overset{N}{\underset{j=i+1}{\sum }}\ldots ..\times \overset{N}{\underset{N=t+1}{\sum }}(r_{\mathrm{ij}}r_{\mathrm{ji}})\times \\ R_{k}R_{l}R_{m}R_{n}R_{o}\ldots ..R_{t}R_{N\;}+\overset{N-2}{\underset{i=1}{\sum }}\overset{N-1}{\underset{j=i+1}{\sum }}\overset{N}{\underset{k=j+1}{\sum }}\ldots ..\times \overset{N}{\underset{N=t+1}{\sum }}(r_{\mathrm{ij}}r_{\mathrm{jk}}r_{\mathrm{ki}}+\\ r_{\mathrm{ik}}r_{\mathrm{kj}}r_{\mathrm{ji}})\times R_{k}R_{l}R_{m}R_{n}R_{o}\ldots ..R_{t}R_{N}+(\overset{N-3}{\underset{i=1}{\sum }}\overset{N}{\underset{j=i+1}{\sum }}\overset{N-1}{\underset{k=j+1}{\sum }}\overset{N}{\underset{l=j+2}{\sum }}\ldots ..\times \\ \overset{N}{\underset{N=t+1}{\sum }}(r_{\mathrm{ij}}r_{\mathrm{jk}}r_{\mathrm{kl}}r_{\mathrm{li}}+r_{\mathrm{il}}r_{\mathrm{lk}}r_{\mathrm{kj}}r_{\mathrm{ji}})\times R_{k}R_{l}R_{m}R_{n}R_{o}\ldots ..R_{t}R_{N})+\\ (\overset{N-2}{\underset{i=1}{\sum }}\overset{N-1}{\underset{j=i+1}{\sum }}\overset{N}{\underset{k=j+1}{\sum }}\overset{N-1}{\underset{l=1}{\sum }}\overset{N}{\underset{m=l+1}{\sum }}\ldots ..\times \overset{N}{\underset{N=t+1}{\sum }}(r_{\mathrm{ij}}r_{\mathrm{jk}}r_{\mathrm{ki}}+\\ r_{\mathrm{ik}}r_{\mathrm{kj}}r_{\mathrm{ji}})\left(r_{\mathrm{lm}}r_{\mathrm{ml}}\right)\times R_{k}R_{l}R_{m}R_{n}R_{o}\ldots ..R_{t}R_{N}+\\ \overset{N-4}{\underset{i=1}{\sum }}\overset{N-1}{\underset{j=i+1}{\sum }}\overset{N}{\underset{k=i+1}{\sum }}\overset{N}{\underset{l=i+1}{\sum }}\overset{N}{\underset{m=j+1}{\sum }}\ldots ..\times \overset{N}{\underset{N=t+1}{\sum }}(r_{\mathrm{ij}}r_{\mathrm{jk}}r_{\mathrm{kl}}r_{\mathrm{lm}}r_{\mathrm{li}}+\\ \left({\;r}_{\mathrm{im}}r_{\mathrm{ml}}r_{\mathrm{ik}}r_{\mathrm{kj}}r_{\mathrm{jii}})\times R_{k}R_{l}R_{m}R_{n}R_{o}\ldots ..R_{t}R_{N}\right)+\\ (\overset{N-3}{\underset{i=1}{\sum }}\overset{N-1}{\underset{j=i+1}{\sum }}\overset{N}{\underset{k=j+1}{\sum }}\overset{N}{\underset{l=j+1}{\sum }}\overset{N-1}{\underset{m=l}{\sum }}\overset{N}{\underset{n=m+1}{\sum }}\ldots ..\times \overset{N}{\underset{N=t+1}{\sum }}(r_{\mathrm{ij}}r_{\mathrm{jk}}r_{\mathrm{kl}\;}r_{\mathrm{li}}+\\ r_{\mathrm{il}}r_{\mathrm{ik}}r_{\mathrm{kj}}r_{\mathrm{ji}})\left(r_{\mathrm{mn}}r_{\mathrm{nm}}\right)\times R_{k}R_{l}R_{m}R_{n}R_{o}\ldots ..R_{t}R_{N}+\\ \overset{N-5}{\underset{i=l}{\sum }}\overset{N-1}{\underset{j=i+1}{\sum }}\overset{N}{\underset{k=j+1}{\sum }}\overset{N-2}{\underset{l=1}{\sum }}\overset{N-1}{\underset{m=l+1}{\sum }}\overset{N}{\underset{n=m+1}{\sum }}\ldots ..\times \overset{N}{\underset{N=t+1}{\sum }}(r_{\mathrm{ij}}r_{\mathrm{jk}}r_{\mathrm{kl}}+\\ r_{\mathrm{ik}}r_{\mathrm{kj}}r_{\mathrm{jii}})(r_{\mathrm{lm}}r_{\mathrm{mn}}r_{\mathrm{nl}}+r_{\ln}r_{\mathrm{nm}}r_{\mathrm{ml}})\times R_{k}R_{l}R_{m}R_{n}R_{o}\ldots ..R_{t}R_{N})+\\ (\overset{N-5}{\underset{i=1}{\sum }}\overset{N}{\underset{j=i+1}{\sum }}\overset{N-3}{\underset{k=i+1}{\sum }}\overset{N}{\underset{l=i+1}{\sum }}\overset{N-1}{\underset{m=k+1}{\sum }}\overset{N}{\underset{n=k+2}{\sum }}\ldots ..\times \\ \overset{N}{\underset{N=t+1}{\sum }}(r_{\mathrm{ij}}r_{\mathrm{ji}})(r_{\mathrm{kl}\;}r_{\mathrm{lk}})\left(r_{\mathrm{mn}}r_{\mathrm{nm}}\right)\times R_{k}R_{l}R_{m}R_{n}R_{o}\ldots ..R_{t}R_{N}+\\ \overset{N-5}{\underset{i=l}{\sum }}\overset{N-1}{\underset{j=i+1}{\sum }}\overset{N}{\underset{k=i+1}{\sum }}\overset{N}{\underset{l=i+1}{\sum }}\overset{N}{\underset{m=i+1}{\sum }}\overset{N}{\underset{n=j+1}{\sum }}\ldots ..\times \overset{N}{\underset{N=t+1}{\sum }}(r_{\mathrm{ij}}r_{\mathrm{jk}}r_{\mathrm{kl}}+\\ r_{\mathrm{ik}}r_{\mathrm{kj}}r_{\mathrm{jii}})(r_{\mathrm{lm}}r_{\mathrm{mn}}r_{\mathrm{nl}}+r_{\ln}r_{\mathrm{nm}}r_{\mathrm{ml}})\times R_{k}R_{l}R_{m}R_{n}R_{o}\ldots ..R_{t}R_{N}) \end{array}$$

where “A” is the square matrix with diagonal elements belonging to normalized values of a material attribute of each hybrid. Equation (3) is the permanent of the decision matrix, which does not contain any complex graph-theoretical parameters. It is the determinant of each matrix with all positive entries. The off-diagonal elements of each matrix are the relative importance rij of attribute i over j, and diagonal elements are the normalized values of attributes for each existing alternative.

## 3. Essential Attributes for Industrial Applications of UHMWPE

As UHMWPE is the gold standard material for several industrial applications, to improve its service life, durability, and impact on the quality of human life, continuous efforts have been in progress since its introduction. For this, UHMWPE blends, composites, and hybrids were prepared to achieve an optimal balance of fundamental requirements for various areas of its application. Although the attributes that are considered in the course of this study may vary for different modification procedures, these are of fundamental importance as far as enhancing the strength of UHMWPE. Therefore, all are briefly discussed in the sub-sections below.

### 3.1. Oxidation Index (OI)

Among the most important parameters or attributes for the UHMWPE- based materials is the oxidation index, which is the quantitative measure of oxidation strength of UHMWPE and/or its composites/blends/ hybrids. It estimates the final product formed as a result of oxidation degradation reactions. According to ISO 5834-2 [30], the oxidation index (OI) is determined by dividing the integrated area of the IR spectra from 1650 cm^−1^–1850 cm^−1^ with the integrating area from 1330 cm^−1^–1396 cm^−1^. The major responsible factor affecting the value of OI is the reaction of free radicals with diffused oxygen within the matrix of UHMWPE, and these free radicals are the precursors of radiation treatment.

### 3.2. Cross-Linking Yield

Another important factor or attribute for UHMWPE-based materials is the cross-linking yield, which is usually enhanced to increase the performance attributes considered in this study. The most sophisticated method to enhance the cross-linking yield of UHMWPE-based materials is treating them with gamma/high energy irradiation. According to the best of our knowledge, the cross-linking yield of UHMWPE/Silane hybrids is almost two times that of a pristine hybrid, which is the main reason for choosing UHMWPE/Silane hybrids in this study. For the estimation of the cross-linking yield, a gel contents (%) measurement is used. The relation for gel contents is:

(5) Gel contents (%)=(w1w0)

where W_0_ and W_1_ are the weights of the sample before and after the extraction of each sample in boiling Xylene for 12 h.

### 3.3. Percent Crystallinity (Xc) and Crystalline Lamellae Thickness (Lc)

Percent crystallinity (Xc) is among one of the most important parameters as far as the utility of UHMWPE based materials in industrial applications is concerned. This is because crystalline centers within the UHMWPE matrix are responsible for the absorption of applied mechanical stress and dissipating it into the surrounding environment via UHMWPE long-chain vibrations. Therefore, an adequate amount of crystalline centers with reasonable thickness are the guarantee for the best performance of UHMWPE-based hybrids in various applications. However, it is a well-established understanding that radiation treatment and/or any modification affect both parameters, i.e., Xc (%) and Lc. For the calculation of both parameters, the following equations, along with DSC data, are used:

(6) Xc=∆H°m∆Hm×100

where ∆Hm and ∆H°m represent the enthalpy of the melting of sample and the enthalpy of melting of perfect crystalline polyethylene (290 J g^−1^), respectively.

Moreover, by using the Thompson–Gibbs equation given below, the crystalline lamellar thickness of the hybrids was also estimated.

(7) Tm=T°m(1−2σLcρc∆Hm)

Here,

- T_m_ is equal to the melting temperature of the hybrid;
- T°_m_ = the equilibrium-melting temperature of 100% crystalline PE is equal to 145.7 °C;
- *ρ_c_* is the crystalline phase density, and its value is equal to 1.005 g/cm^−3^;
- *σ* is the surface energy which is equal to 95.7 × 10^−7^;
- Δ*Hm* is the enthalpy peak area of a 100% crystalline PE and its value is equal to 290 J/g.

### 3.4. Mechanical Characteristics

Mechanical strength is the fundamental requirement for materials used in high-strength industrial applications and is measured in terms of fracture-strain (Eb), yield strength (YS) and, Youngs’s Modulus (YM).

Fracture-strain is the ratio of change in length after test specimen breakage to the initial length, and its optimized value is required for any industrial application. This is because too much cross-linking or too much plasticity has an adverse effect as far as the utility of UHMWPE-based materials is concerned. It is measured in apercentage as follows:

Elongation = ɛ = (Δ*L*/*L*) × 100 (8)

where Δ*L* is the change in length.

The ability of a material to withstand the change in length under applied stress and retain its elastic nature are important performance attributes, and the values comparable to UHMWPE or higher are required for best performance. The formulas for finding these parameters are:

(9) YM=σε=FA ∆LLo=FLoA∆L

All three parameters are calculated from the stress–strain curve obtained from universal tensile testing machines.

## 4. Methodology

For designing any engineering product, the selection of a material is either based on performance or cost; however, from an industrial point of view, the selection is performance-driven because such materials are used to perform a difficult task for a longer period [30]. As the focus of this study is to optimize the structure–property characteristics followed by the ranking of UHMWPE/Silane hybrids subsequent to radiation modification, the major structural attributes of UHMWPE considered in this study are the oxidation index (OI), gel contents (GC), % crystallinity (Xc), and peak melting temperature (T_m_). The performance attributes which are considered in the course of this study are thermal activation energy (E_thermal_), oxidative activation energy (E_oxidation_), fracture-strain (E_b_), crystalline lamella thickness (Lc), yield strength (YS), and Young’s modulus (YM).

### 4.1. Experimental

#### 4.1.1. Materials

For the preparation of UHMWPE/Silane hybrids, vinyltriethoxysilane (VTES), powder form UHMWPE (d = 0.940 g/cm^3^), and acetone (99% pure, i.e., laboratory-grade) were used. All chemicals were purchased from Sigma-Aldrich Chemie, Steinheim, Germany, and used without any further purification.

#### 4.1.2. Hybrid’s Preparations and Modifications

The detailed procedure of hybrid preparation and its modifications can be found elsewhere [5]. Briefly, 0.4 phr of VTES was blended with UHMWPE and pressed into sheets at a temperature of ~150 °C under the pressure of 200 bars while using the automated hot press. After pressing into sheets, hybrids were divided and labeled into the four groups given below:

- For samples labeled as HY-0, 0.4 phr VTES was mixed with acetone then poured into 10 g of UHMWPE powder. The admixture was mixed and dried for further treatment with boiling water for approximately 24 h
- For samples labeled as HY-30, HY-65, and HY-100, 0.4 phr VTES was mixed with acetone then poured into 10 g of UHMWPE powder. The admixture was mixed and dried for further treatment with 30, 65, and 100 kGy of gamma dose, respectively.

The irradiation was conducted in the air using a Co-60 source at a constant dose rate of 1.02 kGy/h, and the choice of dose values was determined due to their importance for implant sterilization and cross-linking. After radiation treatment, samples were tested for structural parameters and performance attributes.

#### 4.1.3. Hybrid’s Characterization

For comprehensive details of experimental investigations, readers are referred to the literature [5,6]. Briefly, a carbonyl (C=O) absorption band of FTIR spectra was used for OI estimation, gel contents were estimated following the ASTM D-2765 standard, the integrated area under the enthalpy peak and the peak position of the endothermic curve obtained from DSC analysis was used for the estimation of Xc (%) and Tm. Furthermore, a stress–strain curve, the Thompson–Gibbs equation [7,31], and the Zhuravlev–Lesokhin–Tempelman equation [32,33] were used for the calculations of fracture-strain (E_b_), yield strength (YS), and Young’s modulus (YM), crystalline lamella thickness (Lc), thermal activation energy (E_thermal_), and oxidative activation energy (E_oxidation_), respectively. A universal tensile testing machine (Model BSS-500 kg, SANS, Transcell Technology, Shenzhen, China) was used to obtain the stress–strain curve, while DSC data was used for estimating the values of crystalline lamella thickness (Lc), thermal activation energy (E_thermal_), oxidative activation energy (E_oxidation_). The measured values of the selected attributes/properties for the optimization and ranking purpose are given in in the results and discussion section.

## 5. Attributes Based Ranking

**Figure 3:**
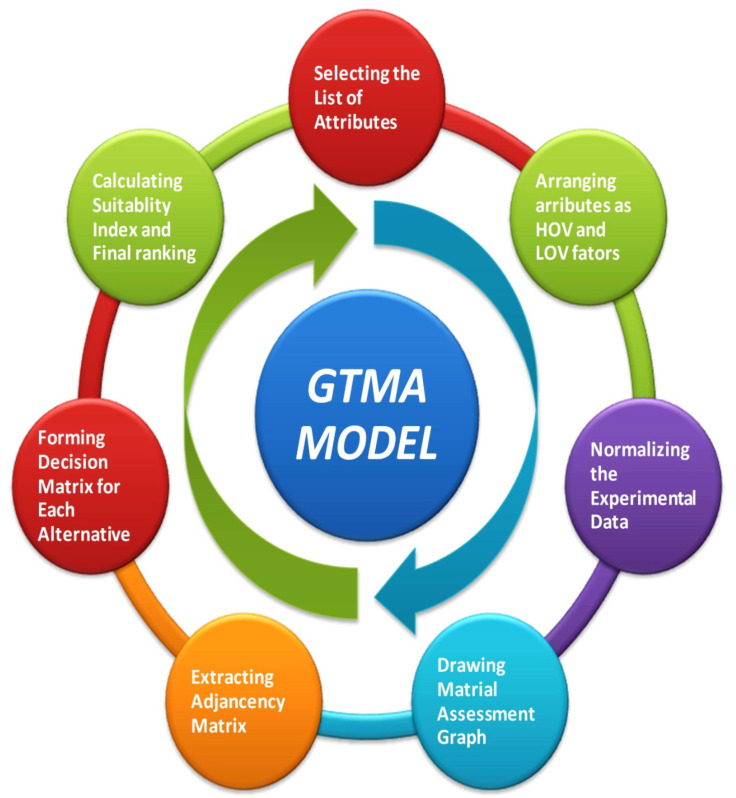
Flow chart representing the stepwise applicability of the proposed GTMA model.

The following flow chart described in Figure 3 summarizes the attribute-based ranking protocol for the study. Figure 3 indicates each step of the method of ranking after experimentations; it is explicitly highlighted in the flow diagram in Figure 3 for a better understanding of the methodology.

## 6. Results and Discussion

The experimental results obtained for all alternatives in this study are tabulated in Table 1.

Table 1 indicates that the synergistic effect of silane and irradiation reduces the free radicals oxidation degradation of UHMWPE via silane grafting extension reactions and siloxane (Si-O-Si) linkages. Although the value of OI is high for irradiated hybrids, on the other hand, the fundamental performance attributes, including onset thermal degradation temperature, peak melting temperature, and crystalline lamellae thickness, E_b_(%), YS, and YM, also need to be considered. Thermal analysis has revealed that irradiated hybrids exhibited a higher onset thermal degradation temperature, peak melting temperature, and crystalline lamellae thickness compared with the water-treated hybrid. In addition, tensile testing has confirmed a 41% and 133% increase in YS and YM values for 100 kGy irradiated hybrids than that of water-treated hybrids. The lower value of OI and double value cross-linking density for 65 kGy irradiated hybrids, along with reasonable enhancement in YM, YS values as compared to other existing alternatives i.e., 30 and 100 kGy irradiated hybrids, make this a potential alternative among existing materials. In short, one cannot rely on single parameters or only on structural parameters, as recently reported by Ahmad A Baksh [28] for choosing the best among existing alternatives. Therefore, the abovementioned parameters are further considered for ranking purposes.

The first step of ranking is the selection/marking of the attribute as an HOV factor or a LOV factor. This is a very important step to determine a suitable choice from existing alternatives. Therefore, understanding the real meaning of HOV and LOV factors is of particular significance. The HOV factor is the one whose highest value is required, while the LOV factor is the one whose lowest value is aimed for the best industrial performance of the hybrid. From a comprehensive (application + material sensitive) literature review, the following key conclusive points are concerned for labeling the attribute as HOV and LOV factors for this particular study:

- Oxidation strength is the first and foremost requirement; therefore, the lowest value of OI and higher value of E_oxidation_ are required. OI is considered as a LOV factor, while E_oxidation_ is labeled as an HOV factor.
- Higher cross-linking yield is the major reason for treating/modifying UHMWPE since its introduction. Therefore, GC (which is ASTM standard for measuring the cross-linking yield) is labeled as an HOV factor.
- The major advantage of UHMWPE-based material is that the mechanical energy, which is usually absorbed through the crystalline phase, is dissipated via its long-chain vibrations, and all-trans interphase regions play the role of transferring the absorbed energy from the crystalline phase to an amorphous one. Therefore, higher contents of crystalline centers are beneficial for the efficient dissipation of absorbed mechanical energy into the surroundings. A higher value of (%) Xc is therefore required, thus justifying its labeling as an HOV factor.
- The importance of T_m_ (°C) and E_Thermal_ (KJ/mol) contributions are negligible and cannot be neglected when considering the long-term service characteristics of UHMWPE-based biomaterials. As a result, in this study, these factors are labeled as LOV factors.
- Usually, crystallite centers are the major source for the dissipation of mechanical energy via long-chain vibrations. In this regard, the mobility of crystalline lamellae for the process of energy dissipation should be higher, which points towards the importance of crystalline lamellae of an adequate thickness for the increased efficacy of UHMWPE as energy dissipaters. In this study, Lc (nm) is included in the list of LOV factors.

**Figure 4:**
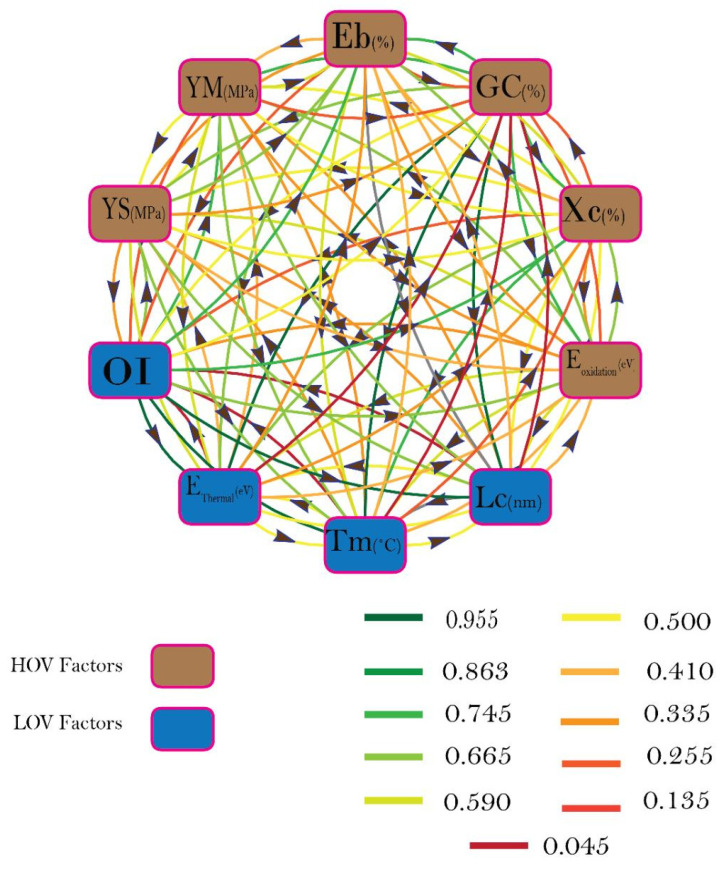
Self-descriptive material selection graph for this study with mentioned HOV and LOV factor and weight–age of relative importance’s among each factor.

After the identification and/or labeling of the HOV and LOV factors, the material selection graph is plotted while keeping in view the relative importance of each attribute/factor. Material attributes and the application-specific strategic literature survey along the 11-scale fuzzy conversation scale, shown in Figure 4, was used for establishing the relative importance of r_ij_ and r_ji_ among the i^th^ and j^th^ factor. After finalizing the graph, the next step was to write the adjacency matrix, as given below, following the steps mentioned in Section 2.

Table 2 indicates quantitative values of structural attributes, i.e., OI, GC, Xc, and T_m_, and performance attributes, i.e., E_thermal_, E_oxidation_, E_b_, Lc, YS, and YM), these are normalized while using their importance as HOV and LOV factors.

Table 3 indicates the normalization of the structures and performance attributes.

**Figure 5:**
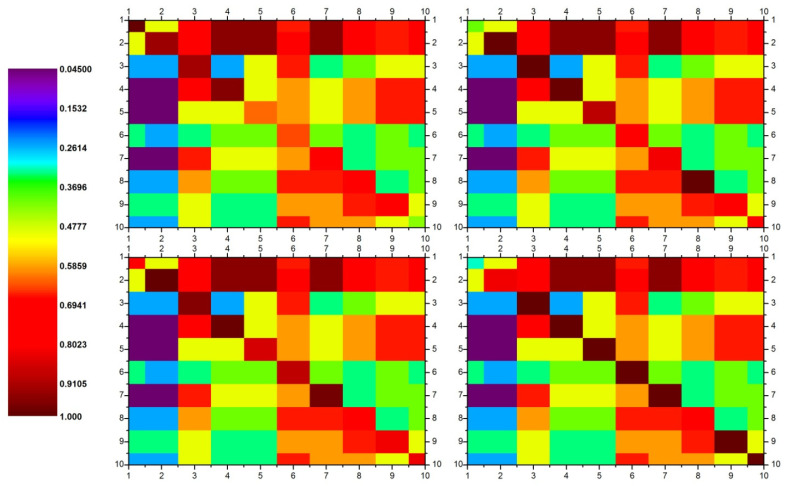
Rainbow color representation of decision matrices for HY-0 (**top right** side), HY-30 (**top left** side), HY-65 (**bottom left** side), and HY-100 (**bottom right** side).

Figure 5 represents the decision of matrices with the help of different colors. Furtherance to it, the normalized values of all hybrids are shown in Table 4.

The most important step after writing down the problem adjacency matrix is to establish decision matrices for each alternative under investigation. In order to accomplish the goal, the normalization of quantitative values of HOV factors and LOV factors given in Table 2 is required. These normalized values of HOV factors for each alternative are calculated by v_i_/v_j_*,* where, vi is the factor quantitative measure for the i^th^ alternative and vj is the factor quantitative measure for the j^th^ alternative, having a larger factor value from the enlisted possible alternatives. It is worth mentioning here that this ratio is only valid for those factors whose higher values are enviable in an application context. For LOV factors (the factors with lower desirable measure), the normalized values are calculated by v_j_/v_i_*,* where vj is the factor quantitative measure for the j^th^ alternative having lower factor-value from enlisted possible alternatives. The quantitative values of structural attributes, i.e., OI, GC, Xc, and Tm, and performance attributes, i.e., E_thermal_, E_oxidation_, E_b_, Lc, YS, and YM), (given in Table 2) are normalized while using their importance as HOV and LOV factors. 

The final step is to write the decision matrix for each alternative/hybrid, to calculate the permanent of each matrix, and to rank the available alternatives. The decision matrices and ranks of all four hybrids are shown in Figure 5. The decision matrices for all four hybrids are shown as color images for better visualization of the difference between the existing alternatives. Each image belongs to the decision matrix of each alternative with HY-0 (top left), HY-30 (top right), HY-65 (bottom left), and HY-100 (bottom right), respectively. The attributes which were under consideration during the course of this study are labeled with numbers 1–10. The diagonal color boxes belong to the normalized values of attributes for each alternative, while the off-diagonal colors represent the strength of correlation among each alternative. It is evident from the intensity of the colors that OI, GC (%), Xc (%), T_m_, and E_thermal_, Eb, YM, YS are the major responsible factors. These factors need to be considered for obtaining an optimum solution for the problems as opposed to the study by Ahmad A Baksh [28], where only three structure parameters, i.e., OI, GC, and Xc (%), were considered for ranking the alternatives.

From the results, it can be seen that HY-65 is the best among the enlisted alternatives. This is the optimized decision, which is made while considering the structural and performance attributes simultaneously. Although the performance attributes YS and YM for 100 kGy hybrids are slightly better, the higher cross-linking yield, OI and E_b_, as given in Table 1, for the 65 kGy irradiated sample is the reason for placing HY-65 as the ranked number one hybrid. The optimization methodology in this paper for selecting suitable alternative for the industrial community is simple and can be extended by including more attributes. Moreover, the methodologies not only give an analysis of the alternatives, but also serves as a visualization of the correlation factors while utilizing the graphical representation. The measurements of the factors and their relative significance are used together to rank the alternatives, and consequently, give a stronger evaluation of the alternatives. Furthermore, using the concept of a permanent matrix is more beneficial for the more accurate evaluation of factors. It contains all possible structural components of the factors and their relative significance, and it, therefore also characterizes the considered selection issue in a more indisputable way.

## 7. Conclusions

Finding a simple and robust computational approach for determining the optimum solution from the existing alternatives of UHMWPE composites from industrial application point of view is the key problem which was addressed in this study. The parametric graph theory and matrix approach were used while considering ten structural and performance attributes. The normalized values of attributes (including oxidation index, gel contents, % crystallinity, peak melting temperature thermal activation energy, oxidative activation energy, fracture-strain, crystalline lamella thickness, yield strength, and Young’s modulus) and their relative importance were used for extracting the decision matrices for each alternative. These decision matrices were then successfully used to obtain the suitability index values and the final ranking of the materials. According to the suitability index value which was obtained from graph theoretical approach hybrid labeled as HY-65, the hybrid irradiated with 65 kGy of gamma dose was ranked as best among existing alternatives and the values of OI, GC (%), and Eb (%) were the major factors for ranking the 65 kGy hybrids as the best choice.

## Figures and Tables

**Table 1:** Measured values of selected attributes/properties for establishing structural–property optimization and ranking of hybrids.

Material Attribute/Property	UHMWPE/Silane Hybrids			
	HY-0	HY-30	HY-65	HY-100
OI	0.17	0.42	0.24	0.54
Gel Contents (%)	84.7	90.1	91	75
Xc (%)	50.1	54.3	52.2	53.9
T_m_ (°C)	137.1	133.3	133.7	131.8
Lc (nm)	10.8	7.5	7.8	6.7
E_oxidation (eV)_	122	133	170	190
E_Thermal (eV)_	446	440	370	361
E_b_ (%)	357	452	360	324
YS (MPa)	21.7	22.7	25.3	30.7
YM (MPa)	422	738	817	984

**Table 2:** Adjacency matrix from the material selection graph (Figure 3) with relative importance among the attributes as off-diagonal elements obtained from 11-point fuzzy logic conversation scale.

Parameters	OI	GC (%)	Xc (%)	T_m_ (°C)	Lc (nm)	E_oxidation (eV)_	E_Thermal (eV)_	E_b_ (%)	YS (MPa)	YM (MPa)
OI	R_1_	0.5	0.745	0.955	0.955	0.665	0.955	0.745	0.665	0.745
GC (%)	0.5	R_2_	0.745	0.955	0.955	0.745	0.955	0.745	0.665	0.745
Xc (%)	0.255	0.255	R_3_	0.255	0.5	0.665	0.335	0.41	0.5	0.5
T_m_ (°C)	0.045	0.045	0.745	R_4_	0.5	0.59	0.5	0.59	0.665	0.665
Lc (nm)	0.045	0.045	0.5	0.5	R_5_	0.59	0.5	0.59	0.665	0.665
E_oxidation (eV)_	0.335	0.255	0.335	0.41	0.41	R_6_	0.41	0.335	0.41	0.335
E_Thermal (eV)_	0.045	0.045	0.665	0.5	0.5	0.59	R_7_	0.335	0.41	0.41
E_b_ (%)	0.255	0.255	0.590	0.41	0.41	0.665	0.665	R_8_	0.335	0.41
YS (MPa)	0.335	0.335	0.5	0.335	0.335	0.59	0.59	0.665	R_9_	0.5
YM (MPa)	0.255	0.255	0.5	0.335	0.335	0.665	0.59	0.59	0.5	R_10_

where R_1_, R_2_,….Ri….R_10_ are the normalized values of each attribute/factor of the respective hybrids under investigation in this study.

**Table 3:** Normalization of structure and performance attributes.

Material Attribute/Property	UHMWPE/Silane Hybrids			
	HY-0	HY-30	HY-65	HY-100
OI	1.00	0.40	0.71	0.31
Gel Contents (%)	0.93	0.99	1.00	0.82
Xc (%)	0.92	1.00	0.96	0.99
T_m_ (°C)	0.96	0.99	0.99	1.00
Lc (nm)	0.62	0.90	0.86	1.00
E_oxidation (eV)_	0.64	0.70	0.89	1.00
E_Thermal (eV)_	0.81	0.82	0.98	1.00
E_b_ (%)	0.79	1.00	0.80	0.72
YS (MPa)	0.71	0.74	0.82	1.00
YM (MPa)	0.43	0.75	0.83	1.00

**Table 4:** Suitability index value (SIV) and ranking of hybrids.

Sample	Suitability Index Value	Ranking
HY-0	2488.43	4th
HY-30	2637.2	3rd
HY-65	3050.24	1st
HY-100	2890.47	2nd

## Data Availability

Data will be provided on demand.
